# Effect and feasibility of non-linear periodized resistance training in people with COPD: study protocol for a randomized controlled trial

**DOI:** 10.1186/s13063-018-3129-y

**Published:** 2019-01-03

**Authors:** Erik Frykholm, Peter Klijn, Didier Saey, Hieronymus W. H. van Hees, Per Stål, Thomas Sandström, Ann Sörlin, François Maltais, André Nyberg

**Affiliations:** 1 Department of Community Medicine and Rehabilitation, Umeå University, Umeå, Sweden; 2 Department of Pulmonology, Pulmonary Rehabilitation Centre Merem, Hilversum, The Netherlands; 3 Department of Pulmonology, Academic Medical Center, Amsterdam, The Netherlands; 4 Centre de recherche, Institut Universitaire de Cardiologie et de Pneumologie de Québec, Faculté de médecine, Université Laval, Québec, QC Canada; 5 Department of Pulmonary Diseases, Radboud UMC, Nijmegen, The Netherlands; 6 Department of Integrative Medical Biology, Umeå University, Umeå, Sweden; 7 Department of Public Health and Clinical Medicine, Umeå University, Umeå, Sweden

**Keywords:** Pulmonary disease, chronic obstructive, Limb-muscle endurance, Limb-muscle strength, Functional performance

## Abstract

**Background:**

In people with chronic obstructive pulmonary disease (COPD), limb-muscle dysfunction is one of the most troublesome systemic manifestations of the disease, which at the functional level is evidenced by reduced strength and endurance of limb muscles. Improving limb-muscle function is an important therapeutic goal of COPD management, for which resistance training is recommended. However, current guidelines for resistance training in COPD mainly focus on improving muscle strength which only reflects one aspect of limb-muscle function and does not address the issue of reduced muscle endurance. The latter is of importance considering that the reduction in limb-muscle endurance often is greater than that of muscle weakness, and also, limb-muscle endurance seems to be closer related to walking capacity as well as arm function than to limb-muscle strength within this group of people. Thus, strategies targeting multiple aspects of the decreased muscle function are warranted to increase the possibility for an optimal effect for the individual patient. Periodized resistance training, which represents a planned variation of resistance training variables (i.e., volume, intensity, frequency, etc.), is one strategy that could be used to target limb-muscle strength as well as limb-muscle endurance within the same exercise regimen.

**Methods:**

This is an international, multicenter, randomized controlled trial comparing the effect and feasibility of non-linear periodized resistance training to traditional non-periodized resistance training in people with COPD. Primary outcomes are dynamic limb-muscle strength and endurance. Secondary outcomes include static limb-muscle strength and endurance, functional performance, quality of life, dyspnea, intramuscular adaptations as well as the proportion of responders. Feasibility of the training programs will be assessed and compared on attendance rate, duration, satisfaction, drop-outs as well as occurrence and severity of any adverse events.

**Discussion:**

The proposed trial will provide new knowledge to this research area by investigating and comparing the feasibility and effects of non-linear periodized resistance training compared to traditional non-periodized resistance training. If the former strategy produces larger physiological adaptations than non-periodized resistance training, this project may influence the prescription of resistance training in people with COPD.

**Trial registration:**

ClinicalTrials.gov, ID: NCT03518723. Registered on 13 April 2018.

**Electronic supplementary material:**

The online version of this article (10.1186/s13063-018-3129-y) contains supplementary material, which is available to authorized users.

## Background

Chronic obstructive pulmonary disease (COPD) is currently the fourth leading cause of mortality worldwide and is expected to become the third by 2020. COPD is now considered a multisystem disease, including limb-muscle dysfunction [1]. Limb-muscle dysfunction is considered one of the most troublesome systemic manifestations of the disease and is characterized by limb-muscle atrophy and by a fiber-type shift toward a less oxidative phenotype, which at the functional level is evidenced by reduced strength and endurance [1, 2]. Importantly, this is negatively associated with clinically relevant outcomes such as exercise tolerance [3], functional capacity [4], daily life activity [5], as well as increased health care service use, dyspnea and poor quality of life [6]. Limb-muscle dysfunction is also closely linked to the prognosis of the disease [7]. For example, a mid-thigh muscle cross-sectional area less than 70 cm^2^ is associated with fourfold increase in mortality after adjusting for age, sex and FEV_1_ (1), while each 10% increment of a quadriceps strength (kg) to body mass index (kg/m^2^) ratio is associated to a 9% reduction in mortality [1, 7].

Limb-muscle dysfunction is a heterogeneous process in patients with COPD [8] but this is rarely considered in the exercise training prescription. To date, if the goal is to improve limb-muscle function, a resistance-training strategy is often recommended before other available exercise modalities for people with COPD [9]. For example, larger effects on muscle strength are obtained with resistance training in comparison to whole body aerobic training or when resistance training is added to an aerobic training protocol [9–11]. However, the optimal resistance training prescription for people with COPD with regard to improving either limb-muscle strength or endurance has yet to be determined [9]. Current guidelines for resistance training in COPD [9] mainly focus on improving muscle strength which only reflects one aspect of limb-muscle function and does not address the issue of reduced muscle endurance. This is important because the reduction in limb-muscle endurance is often greater than that of muscle weakness, approximating 32 to 77% in patients with COPD [1, 2]. Limb-muscle endurance also seem to be more closely related to walking capacity as well as arm function than muscle strength in COPD [11, 12]. Thus, improving limb-muscle endurance in patients with COPD should not be neglected in designing resistance training interventions. Furthermore, resistance training performed in accordance to current guidelines also lack a clear structure on how to individualize training schemes. This is of importance as up to 50% of patients with COPD are considered non-responders after exercise training [13–15]. The consensus is that the same training schedules cannot be used for all subjects, even if the goal is the same. Strategies on how to adapt and vary training schemes based on the individual condition, preferably targeting multiple aspects of the decreased muscle function, is warranted to increase the possibility for an optimal effect for the individual patient [16, 17]. This calls for alternative ways of performing resistance training in COPD.

Periodized resistance training, which represents a planned variation of resistance training variables (i.e., volume, intensity, frequency, etc.), is one strategy that could be used to target muscle strength as well as muscle endurance within the same exercise regimen. In healthy adults, periodized resistance training increases muscle strength as well as muscle power significantly more than traditional non-periodized resistance training (RT) [18]. In people with COPD, non-linear periodized aerobic training has resulted in greater effects on exercise capacity as well as quality of life in comparison to traditional aerobic training [17]. However, the potential effect of utilizing the concept of non-linear periodization of training variables during resistance training in people with COPD is less investigated.

Therefore, we intend to perform a trial to investigate the effect and feasibility of non-linear periodized resistance training (NLPRT) compared to traditional RT for people with COPD.

### Primary objective

1. To compare the effect of NLPRT with RT on isotonic upper (chest press, shoulder flexion) and lower (leg press, calf press) limb-muscle strength and limb-muscle endurance in people with COPD.
  *Hypothesis*: NLPRT will result in a similar effect size with regard to isotonic leg press, chest press, calf press and shoulder flexion muscle strength but in a larger effect size (> 1.16) with regard to muscle endurance than traditional RT at week 9

### Secondary objectives

2. To compare the effect of NLPRT and RT on (1) functional performance measured with the endurance shuttle walk test (ESWT), the 60-s Sit to Stand Test (60STS) and the Unsupported Upper Limb Exercise Test (UULEX), (2) isometric quadriceps muscle strength and endurance, (3) health-related, disease-specific quality of life (HRQoL), (4) dyspnea and (5) the proportion of responders for each of these variables (a response being defined as an improvement that is larger than the known minimal detectable change/or minimal important difference for a given variable).
  *Hypothesis*: NLPRT will result in a greater gain on functional performance, isometric quadriceps muscle endurance, HRQoL dyspnea, as well as a larger number of responders while effects will be similar with regard to isometric muscle strength at week 9
3. To determine the feasibility of NLPRT compared to RT on attendance rate, duration, satisfaction, drop-outs as well as occurrence and severity of any adverse events.
  *Hypothesis*: that NLPRT will be feasible to the same extent as RT over 8 weeks of training
4. To determine the responsiveness of isometric quadriceps strength and endurance measurement to NLPRT and RT.
  *Hypothesis*: isometric quadriceps muscle strength measurements will be highly responsive to NLPRT as well as RT represented by a standardized response mean (SRM) > 0.80, while quadriceps endurance measurements will be highly responsive to NLPRT but moderately responsive to RT (SRM 0.50 > 0.80) at week 9
5. To explore the intramuscular adaptations of NLPRT compared to the intramuscular adaptations of RT
  *Hypothesis*: NLPRT will result in a larger increase in muscle capillarization as well as in the activity of citrate synthase (CS) and lactate dehydrogenase (LDH) compared to RT at week 9

## Methods

**Fig. 1:**
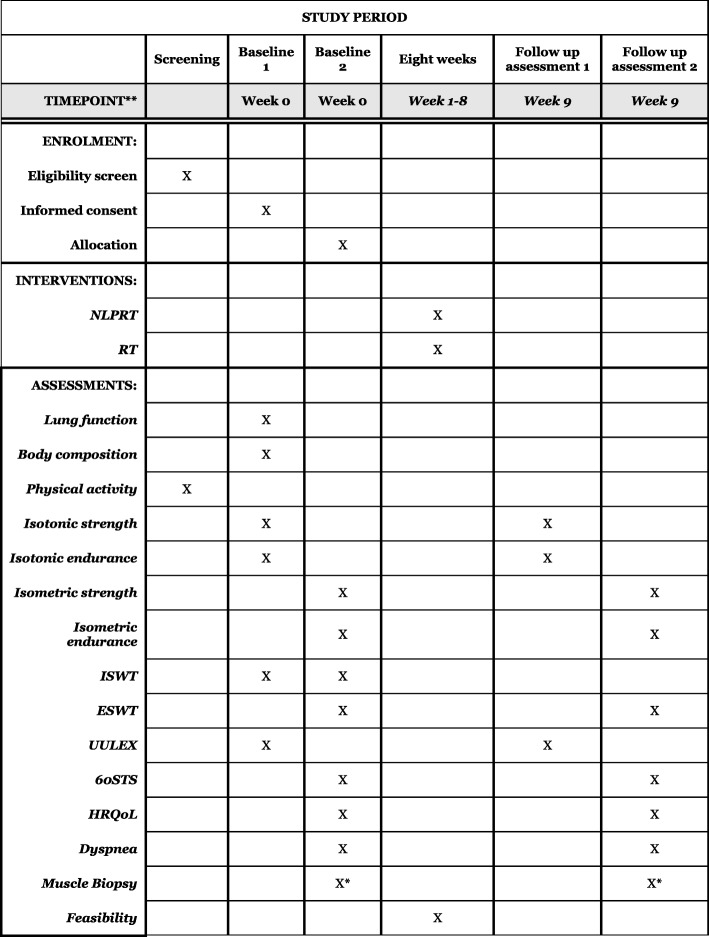
Standard Protocol Items: Recommendations for Interventional Trials (SPIRIT) Figure. Summary of data collection visits. *Performed at two centers (Umeå and Quebec) at a separate third visit during baseline and follow-up assessments. *NLPRT* non-linear periodized resistance training, *RT* resistance training, *ISWT* Incremental Shuttle Walk Test, *ESWT* Endurance Shuttle Walk Test, *UULEX* Unsupported Upper Limb Exercise Test, *60STS* 60-s Sit to Stand Test, *HRQoL* health-related quality of life

**Fig. 2:**
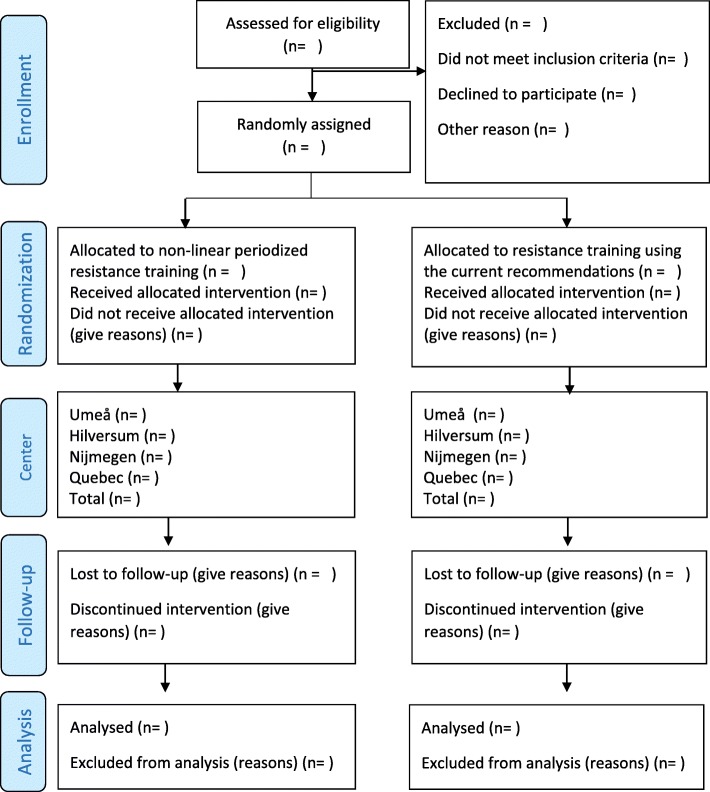
Participant flow diagram

The study is a prospective, assessor-blind, parallel-group, randomized controlled, multicenter trial with a pre- and post-intervention design performed at one Swedish center: Department of Community Medicine and Rehabilitation, Physiotherapy, Umeå University, Umeå, Sweden (site investigators AN and EF), one Canadian center: Institut Universitaire de Cardiologie et de Pneumologie de Québec-Université Laval, Québec City, QC, Canada, (site investigators: DS and FM) and two centers in the Netherlands, Nijmegen (site investigator: WHvH) and at Hilversum (site investigator: PK). The test centers are (or resemble) a training facility for intensive pulmonary rehabilitation with educated staff present at all times. This protocol is reported in line with the Standard Protocol Items: Recommendations for Interventional Trials (SPIRIT) guidelines [19] using the SPIRIT Figure (Fig. 1), participant flow diagram (Fig. 2) and Checklist (Additional file 1) and the trial will be reported according to the Consolidated Standards of Reporting Trials (CONSORT) Statement [20].

### Participants

A total of 64 patients (16 per center) aged between 40 and 89 years with stable moderate to very severe COPD (i.e., post-bronchodilator FEV_1_ < 80% of predicted), will be included.

Exclusion criteria consist of: patients with asthma as primary diagnosis, cardiovascular, neuromuscular, and/or skeletal diseases that are unstable and/or that may contribute to exercise limitation or any other contraindication to exercise; currently participating in a structured exercise or pulmonary rehabilitation program or been involved in pulmonary rehabilitation in the past 6 months; experienced a COPD exacerbation and/or change in medication dosage/frequency in the past 6 weeks. Participants are prohibited to participate in other organized training programs during the trial period but are encouraged to continue with normal everyday physical activity.

### Procedure

Before commencing outcome assessment, all procedures will be explained and the informed consent form will be reviewed and signed. Participants will perform two test visits (three if accepting a muscle biopsy) before the start of the study and immediately following the 8-week intervention period, with adequate recovery between tests and test visits. The same tests and procedures will be used across centers, and pre- and post-intervention assessments will be performed in the same time of the day, with strictly standardized procedures. A summary of study visits and participant data collection schedule are presented in Fig. 1 (SPIRIT Figure), and the flow diagram for the overall study in Fig. 2. Screening and enrollment are planned to start in the spring of 2018 and continue until the target sample size is reached. A 2-year recruitment period is expected. The site investigator at each center will be responsible for the enrollment of participants.

### Sample size calculation

Multicenter trials involve a correlation in data, i.e., participants from the same center are likely to be more similar than subjects from other centers. As such, a correlation potentially affects the statistical power of the trial the sample size needs to be adjusted, often increased, to account for the design of the study [21]. To minimize the effect of using different centers, eligibility criteria as well as design and delivery of training interventions are standardized across centers. Furthermore, in an individually randomized trial that is stratified for centers, similar to the present trial, the design effect is estimated to be < 1 which involves a gain in power, allowing a reduction in sample size rather than an increase [21, 22]. Nevertheless, the sample size calculation is performed with a design effect of 1 and is based on the results from a resistance training meta-analysis on healthy adults in which a group difference of 1.16 (SD 1.09) was seen on muscle function when comparing NLPRT with RT [18]. Thus, based on an expected group difference in effect size of 1.16 (SD 1.09) on muscle function with a power of 80%, two-sided alpha of 0.05 (including 15% drop-outs) 16 patients per group is required (32 in total). However, we plan to recruit a total of 64 patients with an equal distribution of men and women. The planned sample size (*n* = 64, including 15% drop-out) will be sufficient to have power for subgroup analysis based on sex (male and female).

### Randomization and masking

A person not involved with either enrollment, assessments nor training of participants will generate the allocation sequence and assign participants to one of the two interventions.

To prevent foreknowledge of treatment assignment and to keep the allocation sequence concealed from the participants and the researchers, group allocation will be done after the completion of baseline and inclusion tests with a computer random-number generator. Individual randomization, stratified for centers and sex, will be performed with a 1:1 allocation to the intervention and control groups. The allocation sequence will be kept in an opaque, sealed and stapled envelope and will be kept concealed until the end of the outcome assessment. The envelope will be made impermeable to intense light by using aluminum foil inside and sealed using tamper-proof labels.

The outcome assessors will be masked to group allocation. Furthermore, the participants will be given repeated instructions not to reveal their group allocation to the outcome assessors. In case of failure in keeping the outcome assessor masked (i.e., a patient reveals their group allocation), a second trained outcome assessor will be available.

Due to the nature of the intervention neither participants nor instructors of the training sessions will be masked during the intervention, the hypotheses of the trial will, however, not be revealed to the participants before and during the trial. The data analysts will be masked to group allocation.

## Training interventions

Participants will be randomized to either NLPRT or RT of leg press, shoulder flexion, calf press and chest press exercises (three times per week for 8 weeks). The following will be similar for both the NLPRT and the RT groups.

1. All exercises will be performed using training equipment available at each participating center. Leg press, chest press and calf press exercises will be performed using available weight machines and the shoulder flexion exercise will be performed using free weights
2. Exercise order will be pre-determined: (1) leg press, (2) shoulder flexion, (3) calf press and (4) chest press. This exercise order will be used in order to alternate between lower and upper-limb exercises and between multiple- and single-joint exercises. All exercises will be performed bilaterally
3. Rest between exercises will be standardized to 4 min and all exercises will be performed using dynamic repetitions with both concentric and eccentric muscle actions. The speed of motion in each exercise will be controlled by the breathing of the participant, we expect a moderate exercise velocity, i.e., 1–2:1–2 s in the concentric and the eccentric phase, respectively
4. All sessions will be supervised and conducted by local professionals using a group format, with approximately two to four participants per group
5. Each session will span approximately 60 min including a mandatory 5-min rest before and after the training sessions. During this time, oxygen saturation, heart rate, dyspnea and general fatigue (rated on the Borg revised category ratio 0–10 scale (Borg CR10) [23]), will be collected
6. Dyspnea, limb-muscle fatigue and exertion ratings on Borg CR10, the later defined as “how heavy the exercise felt,” will be collected immediately after each set of exercise
7. Exercise volume for each training session = (number of repetitions per set × sets per exercise × exercises per session) will be reported
8. New 1 repetition max (RM) and multiple RM tests will be performed three times during the 8-week intervention period (after session 5, 10 and 15) in order to optimize exercise loadings. If a 1-RM test is lower than the prior 1 RM or if there is a mismatch between calculated training load from the new 1 RM and training load during the previous week, the higher training load will be used and a new test will be performed prior to the next training session to minimize the impact of alterations in daily fitness
9. No general warm-up will be performed prior to sessions but a specific warm-up set of 15 repetitions using a load corresponding to 30% of 1RM prior to each exercises will be performed within the NLPRT and RT sessions

### NLPRT

**Fig. 3:**
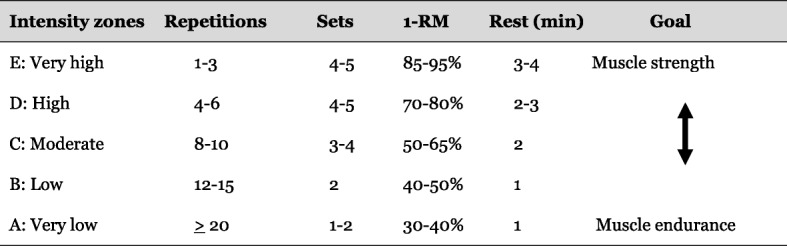
Structure for periodized resistance training [24]

The primary objectives of the NLPRT program will be to increase muscle strength as well as muscle endurance. The NLPRT program will be designed based on the structure shown in Fig. 3. The 8-week NLPRT program will target various aspects of limb-muscle function by alternating between the different intensity zones [24]. Number of sets, number of repetitions, intensities (% of 1 RM) and rest periods will be based on what is presented in Fig. 3.

**Fig. 4:**
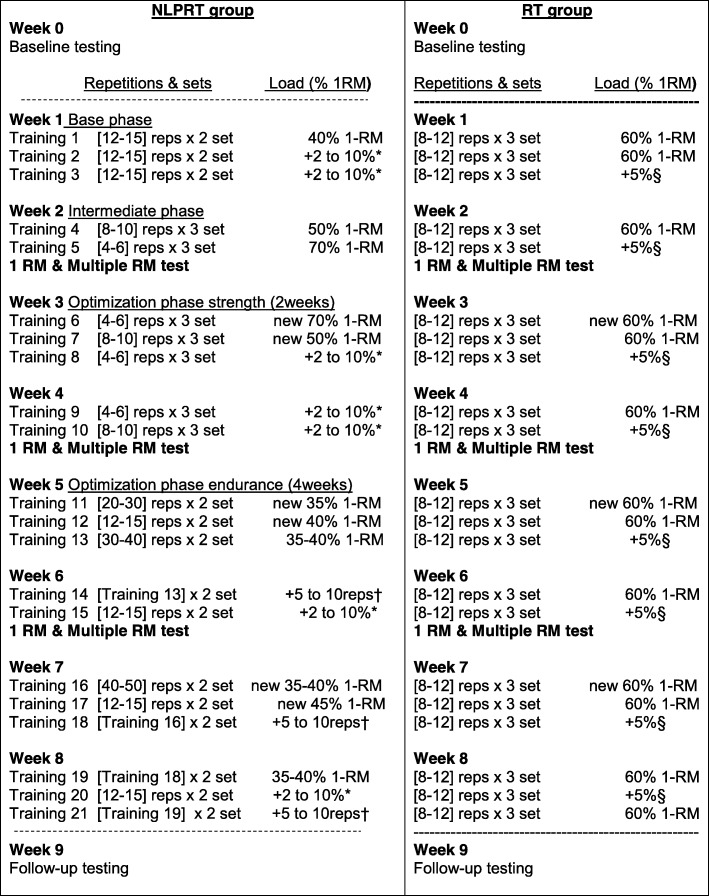
Pre-planned setup of the non-linear periodized resistance training (NLPRT) and resistance training (RT) program. *For < 20 repetition sessions, adjustments will result in increases in intensity ranging between 2 and 10% [23]. †For ≥ 20 repetition sessions, adjustments will result in increases in volume (+ 10 repetitions for lower extremity exercise and + 5 repetitions for upper extremity exercises). §A fixed increase in load of 5% will be added if 12 repetitions are performed in all sets in 2 consecutive sessions [9, 26]

Furthermore, the NLPRT program will be divided into three phases, a base phase (1 week), an intermediate phase (1 week) and an optimizing phase (6 weeks (of which 2 weeks are optimized for limb-muscle strength and 4 weeks optimized for limb-muscle endurance)). An overview of the pre-planned NLPRT setup, including information on how exercises will be progressed, is seen in Fig. 4. Within the NLPRT program, the first 4 weeks will focus more on muscle strength (exercise zones C and D) while the last 4 weeks will focus more on muscle endurance (exercise zones A and B). Progression of exercise will be symptom dependent and will be based on Borg CR10 exertion and fatigue ratings.

### RT

The primary objective of the RT group will be to increase muscular strength. The RT program will be performed in line with current guidelines, i.e., three sets of 8–12 repetitions at an initial load equal to 60% of 1RM (similar to zone C [Fig. 3]) [9, 25] that are recommended for increasing muscle strength in patients with COPD [9]. Rest between sets will be 2 min. Progression of exercises, fixed 5% increase in load, will be performance-based, i.e., an increase occurs when an individual can perform 12 repetitions on the current workload, on two consecutive training sessions [9, 26]. An overview of the pre-planned structure for the traditional resistance training group is seen in Fig. 4.

#### Adherence to intervention

To promote participant retention, efforts will be made to make training sessions in both groups enjoyable for participants. This will include easy access to the facility by the means of transportation preferred by the participants, small training groups in a comfortable setting using equipment suitable for this group of people, and personal monitoring from medically educated instructors. To promote the number of sessions attended during the intervention period, the intervention period can be extended by maximum 1 week due to missed sessions.

The adherence to the intervention will be documented by the instructor conducting the training sessions. In the case that a participant misses a training session without prior notice, the instructor will contact the participant by phone on the same day.

#### Participant safety

Reasons for immediately stopping an outcome tests or training session for people with COPD will include the following: (1) chest pain, (2) intolerable dyspnea, (3) leg cramps, (4) staggering, (5) diaphoresis and (6) pale or ashen appearance [27]. The assessors and instructors are trained to recognize these problems and to deliver the appropriate intervention. Further, this will be reported as an adverse event. If a participant experiences pain/discomfort from the exercising limb during training no progression will occur even if the participant reaches other requirements for progression in the NLPRT or RT group, respectively. If pain prohibits execution of an exercise, this specific exercise will be removed.

#### Data collection, management and analysis

The assessors will have previous experience of the included outcomes and formal training will be provided to the instructors of the training programs. If necessary, protocols for each measurement and exercise will be developed to assist instructors. These protocols will be available on request from the principal investigator.

##### Screening tests and baseline characteristics

A pulmonary function test (spirometry), while being on the participant’s usual bronchodilator therapy, will be obtained according to guidelines [28]. Anthropometrics will include height, weight and body mass index. To document baseline level of physical activity, participants will also be instructed to wear an accelerometer (DynaPort®, McRoberts BV, The Netherlands), placed on the lower back during seven consecutive days before the intervention period. The quantity of physical activity will be assessed using the median number of steps per day collected on at least two consecutive weekdays over at least 8 h of daytime [29]. Primary and secondary outcome assessments will be conducted at two (three if accepting a biopsy) test occasions at weeks 0 and 9 (before and after intervention). Before and directly after all physical tests, the perception of dyspnea and muscle fatigue will be measured with the Borg CR10 [23] as commonly and reliably used in patients with COPD [30–32].

### Primary outcomes

#### Isotonic strength and endurance

Isotonic maximal strength (1 repetition maximum (1RM)) and endurance test (maximum number of repetitions performed at 45% of 1 RM) will be performed during leg press, shoulder flexion, calf press and chest press using a procedure earlier used in an untrained middle-aged population [33]. All RM tests will be separated by a 5-min rest period.

All tests will be performed using exercise equipment that are available at each included center. Leg-press, chest-press and calf press tests will be performed using available weight machines and the shoulder flexion test will be performed using free weights. The tests will be performed in order to individualize progression and to determine the effects of the training performed after the 8-week intervention period. 1RM is defined as the maximum load able to be lifted with good technique through the full range of motion (ROM) [34]. The structure of all 1RM testing is designed so that a 1RM could be achieved in 3–5 attempts. All 1RM test will be preceded by a warm-up of 10 repetitions, using a light load, performed from the start position to the end position in each exercise. The start loading for 1RM testing will correspond to a perceived resistance of 50–70% of 1RM with gradual increases in each trial until 1RM is achieved. The rate of the gradual increase in load is dependent on the participant’s self-perceived capacity and 3–5 min of rest will be used between trials. Lastly, the settings and load of each exercise trial will be noted in the protocol.

- Leg press will be tested with the participants placed in a seated leg-press machine, adjusted so that the range of movement (ROM) of the knee joint will be from 90° flexion to full extension without discomfort in hip or ankle joints. Feet will be positioned shoulder-width apart and fully in contact with the surface during the whole ROM and arms held crossed over the chest. The start position for the test will be with the knee joint in 90° flexion. The placement of the feet will be noted so that the pre- and post-intervention testing will be done in the same position
- Shoulder flexion will be tested using dumbbells, performed while seated on a bench with backrest (120° hip angle) with both feet on the floor. Using one dumbbell in each hand, the participant starts with arms hanging towards the floor, then raises the dumbbell, thumbs pointing upwards, with a fully extended elbow using flexion of the shoulder in the sagittal plane to a 120° shoulder flexion, defined by when the participant can see their own elbow, and returns to the start position. A string will be attached in front of the participant to help participants as well as evaluators to ensure that the movement will be performed in the whole ROM
- Calf press will be tested using the same machine as for the leg press. The feet are placed so that when the participant’s knees are fully extended the legs are aligned with the applied force from the machine. The starting position will be with fully extended knees and the whole sole of the feet on the surface and the end position will be at full individual plantar flexion. The placement of the feet will be noted so that the pre- and post-intervention testing will be done in the same position
- Chest press will be tested with the handlebars placed at mid-sternum height and while the participant grips the handles with the elbows at 90° flexion at sternum height. The back should lie be firmly on the backrest and both feet on the floor. When the participant reaches full extension in the elbows, the end position of the movement will be considered reached

For the multiple RM (limb-muscle endurance) test, the settings and starting positions from the 1RM test will be used. Our multiple RM is defined as the maximum number of repetitions able to be lifted with a good technique through the full ROM at an intensity corresponding to 45% of 1 RM [34]. In order for training specificity, an upper limit of 60 repetitions on the multiple RM test will be used at baseline testing. If a participant exceeds 60 repetitions on a multiple RM test at baseline, the test will be redone with a higher resistance. A maximum of three tests will be performed. Test order will be pre-determined: (1) leg-press, (2) shoulder flexion, (3) calf press and (4) chest press. Warm-up will be one set of 10 repetitions using 50% of the resistance to be used in the multiple RM test. The pace will be controlled by the participant’s breathing.

### Secondary outcomes

#### Functional performance

Walking performance will be evaluated by the endurance shuttle walk test (ESWT) performed in accordance to guidelines, reported in meters or seconds [35, 36]. The incremental shuttle walk test (ISWT) will be performed in order to determine the correct walking speed for the ESWT. The ISWT continues until the participant can no longer continue or cannot keep up with the required pace. The maximum duration of the test is 20 min [35]. The ESWT is a derivative of the ISWT, where patients walk for as long as possible at a predetermined percentage of maximum walking performance as assessed by the ISWT [36]. One test is sufficient to obtain a reliable measure [35]. The ESWT are responsive to changes with interventions in patients with COPD [37].

During the 60STS, participants will be instructed to fully stand up and sit down as fast as possible, as many times as possible within 60 s. Arms will be held folded across the chest with feet remaining in full contact with the floor. The number of repetitions performed within 60 s will be collected. Chair height will be 48 cm or low enough to allow both feet to be in full contact with the floor. The test is reliable, valid and responsive for measuring functional exercise capacity for people with COPD [38, 39].

The UULEX test is performed seated. Start position of the test is seated, with the plastic bar (0.2 kg) held with both hands at shoulder width, resting on the proximal parts of the thighs of the participant. Participants will then be asked to raise it from hip to the UULEX eight-level chart for 2 min at the first level and thereafter, 1 min at each level with a cadence of 30 movements per min. If a patient reaches the highest level, the plastic bar will be replaced by a heavier one every min. There are five different bar weights (0.2, 0.5, 1, 1.5, 2 kg) and participants will continue on the highest level until symptom limitation [40].

#### Isometric quadriceps strength and endurance

In addition to isotonic measurements of muscle strength and endurance, we will also perform isometric measurements, since the different techniques (isotonic versus isometric) might differ with regard to clinical relevance and responsiveness to training [41].

Isometric testing will be performed either using a Biodex Multi-Joint System 3 or 4 (Biodex Corp., Shirley, NY, USA) or a fixed-strain gauge (Biopac System inc, Goleta, CA, USA), depending on the availability of system at each respective center.

After a warm-up of two to three submaximal contractions, five maximal trials will be performed with each contraction sustained for 5 s. One-minute rests will be given between repeated trials to minimize muscle fatigue. Quadriceps maximal voluntary contraction (MVC) torque will be recorded in Newton meters (Nm) for each contraction. Thus, for strain-gauge measurements the Nm will be calculated by dividing force in Newtons by the length of the lever arm. The peak of the two best reproducible contractions (within a 5% coefficient of variation) will be reported as the test result.

After a 15-min rest period, the muscle strength test will be followed by an isometric measurement of quadriceps endurance. Participants will be instructed to maintain a tension representing 60% of their MVC until exhaustion. A computer screen will serve as a feedback mechanism to help subjects maintain the determined submaximal tension. Subjects will be strongly encouraged to pursue until tension drop under 50% MVC. The time to fatigue, defined as the time at which the isometric contraction reached 50% MVC, will be used as a measure of muscle endurance.

#### HRQoL

The self-administered version of the chronic respiratory disease questionnaire (CRQ-SA) [42] will be used to assess HRQoL. The CRQ is a widely used disease-specific questionnaire to assess symptoms of people with COPD. The CRQ is valid and responsive to treatment [43], and has previously been used to evaluate the effects of resistance training [31, 44].

#### Dyspnea

The Borg CR10 will be used to quantify the level of dyspnea during exercise training and tests while general dyspnea in daily life will be evaluated using the dyspnea subscale of the CRQ.

#### Number of responders

The total amount of responders, i.e., defined as a response over the known minimal detectable change/or minimal important difference for included test(s), will be determined and compared. For example, the minimal important difference after rehabilitation is suggested to be 177 s for the ESWT [45] and three repetitions for the 60 STS [46].

#### Feasibility

Feasibility of the training programs will be assessed and compared between groups from the attendance rate, duration, satisfaction, drop-outs as well as occurrence and severity of any adverse events.

- Attendance rate will be evaluated by calculating the number of attended sessions divided by total number of sessions
- Duration of exercise sessions will be evaluated by measuring the time taken to complete an exercise session, including the 5-min mandatory rest periods before and after the exercise session
- Patient satisfaction with the exercise regimens will be recorded by adapting an existing patient satisfaction questionnaire previously used for single-legged cycling [47].
- The number of drop-outs will be collected and compared across resistance regimens
- Information about adverse events will be collected for both exercise protocols. Two independent pulmonologists and one physiotherapist whom will not be involved in the study will evaluate the adverse events. The severity of the adverse events will be assessed and rated into four different categories: (1) minor and temporary, (2) serious symptoms (potential risk of severe injury or life threatening), (3) manifest injury or disease and (4) death, as previously used [48]. An adverse event rate will be calculated for each patient as the total number of sessions during which any adverse events occurred divided by the total number of attended sessions

#### Intramuscular adaptations

Vastus lateralis muscle biopsies are sampled in Umeå (10–16 pre/post biopsies) and in Quebec (10–16 pre/post biopsies) to assess muscle fiber size, expression of slow and fast subtypes of contractile myosin heavy chain isoforms in fibers, oxidative (CS, EC 4.1.3.7) and glycolytic (LDH, EC 1.1.1.27) enzyme activities as well as capillarization (capillary density, number of capillaries around fiber and capillaries around fibers relative to its cross-sectional area).

Possible correlations between capillary parameters, fiber phenotype composition, fiber cross-sectional area, enzymatic activity and other outcome measures, such as quadriceps muscle strength and endurance, after the resistance training interventions will be evaluated.

### Data management

All data will be coded and reported on a group level. It will not be possible to identify specific individuals in the trial. The participants’ identities will only be known by the research group at respective center and the principal investigator.

The REDCap (Research Electronic Data Capture) Survey, a secure, Health Insurance Portability and Accountability Act compliant, web-based survey application will be used to manage and store project data [49]. To ensure confidentiality, data dispersed to project team members will be blinded of any identifying participant information. All records that contain names or other personal identifiers, such as locator forms and informed consent forms, will be stored separately from study records identified by code number at local research centers, respectively. All local databases will be secured with password-protected access systems.

### Statistics

The primary analyses will be an intention-to-treat analysis (including all participants randomized) in addition, a complete case population (participants with complete outcome measurements independent on adherence to intervention), and a per-protocol analysis (defined as at least 80% overall attendance rate as well as no exacerbations during the last 2 weeks prior to follow-up assessment) will be performed. Missing data will be imputed in the intention-to-treat analysis using multiple imputation assuming data is missing at random conditional on participant severity of disease and history of exacerbations. This is because severity of disease and history of exacerbations are known risk factors for future exacerbations and may affect adherence to the training protocol [28].

Mixed models will be used for analysis of data with individuals at level 1 and center at level 2. Estimates of effect sizes will be computed using Cohen’s *d* (*d* = difference in group means/error SD within). Calculated as the difference between predicted means from the final mixed-effects model for a given pair of groups divided by the estimated within-group error SD in the model with the estimated value of $2{\sigma}_e^2$, where ${\sigma}_e^2$ is the residual variance. To judge the quality of the model we will analyze the residuals. For responsiveness, values of 0.20, 0.50 and ≥ 0.80 correspond to small, moderate and large responsiveness, respectively [50].

We will consider two-sided *p* values < 0.05 as statistically significant for the primary outcomes without correction for a multiplicity of tests assuming that each test represents an independent function based on the specificity principle of training [26]. Relationships between parameters will be evaluated using Pearson correlation coefficients. The strength of the correlation coefficients will be categorized as low (0–0.25), moderate (> 0.25–0.50), strong (> 0.50–0.75), very strong (> 0.75). Pre-planned subgroup analyses are differences between men and women. Statistical analysis will be performed using the statistical package SPSS 23.1.

### Amendments

Any modifications to the protocol which may impact on the conduct of the study, potential benefit of the patient or may affect patient safety, including changes of study objectives, study design, patient population, sample sizes, study procedures or significant administrative aspects will require a formal amendment to the protocol. Such amendments will be agreed upon by the research group with the final decision by the principal investigator, and, if needed, approved by the Local Ethics Committees.

Administrative changes of the protocol (e.g., minor corrections and/or clarifications) that have no effect on how the study is conducted will be agreed upon by the research group with the final decision by the principal investigator and documented and presented upon publication.

## Discussion

Improving limb-muscle function is an important therapeutic goal in the management of patients with COPD for which resistance training often is recommended over other available exercise training modalities [8–10]. Even so, the optimal resistance training prescription for people with COPD remains to be determined [8]. Among healthy adults, periodized resistance training has been found to increase limb-muscle strength significantly more than traditional RT [17]. Periodization, which represents a planned variation of resistance training variables, could be done in several different ways, e.g., using either linear-, block- or non-linear, (undulated) periodization strategies. In people with COPD, the latter strategy has been shown to lead to larger benefits in comparison to traditional, non-periodized training when applied during aerobic exercise training [16]. However, whether the concept of non-linear periodization of training variables would be beneficial also during resistance training in people with COPD remains to be determined.

The proposed trial will provide new knowledge to this research area by investigating and comparing the feasibility and effects of NLPRT compared to traditional RT. If, as we hypothesize, NLPRT produces larger physiological adaptations than RT, this project may influence the prescription of resistance training in patients with COPD. The NLPRT approach used within the present project is not only designed to optimize the effects of exercise training on limb-muscle strength, but also on limb-muscle endurance by targeting multiple aspects of limb-muscle function commonly seen among patients with COPD [1]. Furthermore, the present project will also include assessment of potential intramuscular adaptations following two different resistance training approaches. This is highly warranted [51] and should provide novel insight in understanding the role of resistance training as a countermeasure of limb-muscle dysfunction in COPD. The results of this project will also provide novel information about the feasibility of utilizing the concept of NLPRT in clinical settings, potentially providing a highly effective and feasible resistance-training strategy that could be easily implemented in clinical practice.

### Trial registration

The clinical trial has been registered before the enrollment of the first participant. Date of trial registration: 13 April 2018. ClinicalTrials.gov identifier: NCT03518723. The recruitment will begin in 2018 and will continue until sufficient power is reached.

### Trial status

The trial is in the phase of recruiting participants at the time of submission of this protocol on 9 May 2018.

## Additional file

Additional file 1: Standard Protocol Items: Recommendations for Interventional Trials (SPIRIT) 2013 Checklist: recommended items to address in a clinical trial protocol and related documents. (DOC 120 kb)

## Abbreviations

1-RM: One repetition max
60STS: The 60-s Sit to Stand Test
Borg CR10: Borg revised category ratio 0–10 scale
CONSORT: Consolidated Standards of Reporting Trials
COPD: Chronic obstructive pulmonary disease
CRQ-SA: The Self-administered version of the Chronic Respiratory Disease Questionnaire
ESWT: Endurance Shuttle Walk Test
FEV_1_: Forced expiratory volume in 1 s
HRQoL: Health-related quality of life
ISWT: Incremental Shuttle Walk Test
Multiple RM: Multiple repetition max
MVC: Quadriceps maximal voluntary contraction
NLPRT: Non-linear periodized resistance training
RT: Non-periodized resistance training
SPIRIT: Standard Protocol Items: Recommendations for Interventional Trials
SRM: Standardized response mean
UULEX: Unsupported Upper Limb Exercise Test
